# Cluster B personality disorders and its associated factors among psychiatric outpatients in Southwest Ethiopia: institutional-based cross-sectional study

**DOI:** 10.1186/s12888-022-04143-3

**Published:** 2022-07-26

**Authors:** Muzeyen Jemal, Worknesh Tessema, Liyew Agenagnew

**Affiliations:** 1 College of Health and Medical Science, Department of Psychiatry, Mettu University, Mettu, Ethiopia; 2 Institute of Health, Faculty of Medicine, Department of Psychiatry, Jimma University, Jimma, Ethiopia

**Keywords:** Cluster B personality disorders, Mental illness, Outpatients, Jimma, Ethiopia

## Abstract

**Background:**

Diagnosis of co-occurring personality disorders, particularly the most comorbid cluster B personality disorders in psychiatric patients is clinically important because of their association with the duration, recurrence, and outcome of the comorbid disorders. The study aimed to assess the prevalence of cluster B personality disorders and associated factors among psychiatric outpatients in Jimma Medical Center.

**Methods:**

An institution-based cross-sectional study was conducted among 404 patients with mental illnesses at Jimma Medical Center from July 15 to September 14, 2021. A systematic random sampling method was used to recruit the participants. Personality disorder questionnaire four (PDQ-4) was used to assess the prevalence of cluster B personality disorders through a face-to-face interview. Data were entered into Epi Data Version 4.6 and exported to SPSS Version 26 for analysis. Logistic regression analysis was done and variables with a *p*-value less than 0.05 with a 95% confidence interval in the final fitting model were declared as independent predictors of cluster B personality disorders.

**Result:**

Amongst 401 respondents with response rate of 99.3%, slightly less than one-fourth (23.19%, *N* = 93) were found to have cluster B personality disorders. Unable to read and write(AOR = 3.28, 95%CI = 1.43—7.51), unemployment(AOR = 2.32, 95%CI = 1.19—4.49), diagnosis of depressive (AOR = 3.72, 95%CI = 1.52–9.10) and bipolar-I disorders (AOR = 2.94, 95%CI = 1.37—6.29), longer duration of illness (AOR = 2.44, 95%CI = 1.33—4.47), multiple relapses (AOR = 2.21, 95%CI = 1.18–4.15)), history of family mental illnesses (AOR = 2.05, 95%CI = 1.17—3.62), recent cannabis use (AOR = 4.38, 95%CI = 1.61—11.95), recent use of alcohol (AOR = 2.86, 95%CI = 1.34—6.10), starting to use substance at earlier age (AOR = 4.42, 95%CI = 1.51 -12.96), and suicidal attempt (AOR = 2.24, 95%CI = 1.01—4.96), were the factors significantly associated with cluster B personality disorders.

**Conclusion:**

The prevalence of cluster B personality disorders was high among mentally ill outpatients and found to be important for mental health professionals working in the outpatient departments to screen for cluster B personality disorders as part of their routine activities, particularly those who have mood disorders, longer duration of illness, multiple relapses, history of family mental illnesses, suicidal attempt and are a current user of alcohol and cannabis.

## Background

Personality disorders (PDs) are defined as “an enduring pattern of inner experience and behavior that deviates markedly from the expectations of the individual's culture, is pervasive and inflexible, has an onset in adolescence or early adulthood, it is stable over time, and leads to distress or impairment”. It is categorized into three clusters (A, B, and C), personality changes due to another medical condition, other specified personality disorders, and unspecified personality disorders. Cluster B or dramatic cluster consists of 4 subtypes, which are antisocial, borderline, histrionic, and narcissistic PD [1]. They are excessively demanding, manipulative, emotionally unstable, interpersonally inappropriate, and may attempt to create relationships that cross professional boundaries that place physicians in difficult or compromising positions [2].

Cluster B PDs are the most common personality disorders in clinical settings and are characterized by severe functional impairments, substantial treatment utilization, and a high mortality rate by suicide, which is 50 times higher than the rate in the general population [3, 4]. People with these disorders present with psychosocial functioning problems, suicidal behaviors, and more psychiatric comorbidities especially with other personality disorders, substance misuse, and other axes I conditions [5–7]. Patients with axis-I and cluster B PDs comorbidity presented with an earlier onset, more severity in suicide attempts, hospitalizations, self-harm behaviors, and accounting for more impairment in functioning than patients only with axis-I disorders [3, 8]. Many studies reported that factors like unemployment, mood disorders, a history of family mental illness, number of relapses and admissions, substance abuse, and suicidal behaviors were directly associated with cluster B PDs [9–11], while factors like age and educational level were inversely related [7].

Lastly, cluster B PDs is a chronic condition associated with a multitude of medical and social problems [12] and becomes increasingly common in mental health services, the judicial system, and prison settings [13]. Therefore, diagnosing a co-occurring personality disorder in psychiatric patients with another disorder is clinically important because of their association with the duration, recurrence, and outcome of comorbid disorders [14].

Studies of the frequency and correlates of PDs should be replicated in clinical populations where the disorder and comorbidity rates are higher to provide the clinicians with information that has more direct clinical uses [15, 16]. Cluster B personality disorders are the most frequent among outpatients [16, 17]; have the highest prevalence of any co-occurrence with other mental illnesses (83.8%) with a predominance of mood disorders (48.8%) [17].

Globally, the overall prevalence estimate of cluster B personality disorders among mentally ill outpatients was 23% [18], ranging from 9.8% [19] to 66.7% [20]. It influences the prognosis, costs, and response to treatment of many clinical syndromes; increases morbidity, and mortality of the patients, and is significantly associated with global, cognition, and social interaction impairments [19, 21, 22]. Moreover, personality disorders are a predisposing factor for many other psychiatric disorders, including substance use disorders, suicide, mood disorders, impulse-control disorders, eating disorders, and anxiety disorders [21].

Despite the aforementioned importance in diagnosing this disorder, little attention is given by the clinicians in their daily activities [3] and they are sometimes reluctant to diagnose them [23], especially in developing countries. Finally, since PD is by its nature ego-syntonic [21], most of the patients present for treatment fail to complain to their clinician. Due to this it is underdiagnosed and got very less consideration in the treatment, despite its great contribution to social and functional impairment, adverse outcomes like more prolonged hospitalization, episodes of illnesses, and high direct costs through high utilization of healthcare systems.

Almost all of the studies done in this area are from developed countries and most of them are done on subclinical populations. Thus, detection and treatments of those disorders among psychiatric outpatients are far-reaching significances to minimize adverse outcomes and reduce mortality and morbidity associated with it, especially in developing countries, where data is not available currently in the study area. Recognizing the magnitude of the problem is important for designing early and appropriate intervention; to reveal health professionals’ insight into the prevalence of cluster B PDs which is vital for identifying treatment needs and for the provision of psychiatric services. Also, it will be helpful to understand patients with this problem and improve their quality of life by addressing their problems accordingly. Moreover, it will lay a background for further studies and will be added to the limited body of literature on the prevalence of cluster B PDs in the developing region. Thus, the overall aim of this study was to assess cluster B PDs and associated factors among mentally ill patients attending outpatient treatment at Jimma medical center (JMC).

## Methods and materials

### Study design and setting

The institution-based cross-sectional study was conducted from July 15 to September 14, 2021, at JMC. JMC is found in Jimma town, Oromia regional state, 352 km far from Addis Ababa to the southwest. JMC is one of the oldest governmental hospitals, which was established in 1937 during the Italian occupation for the service of their soldiers. Since then, it has been running as a public hospital under the ministry of health by different names, currently named “JMC” and gave services to about 15 million populations in southwest Ethiopia. The psychiatric clinic of JMC was established in 1996 next to Amanuel mental health specialized hospital. Currently, more than 1000 patients are attending follow-up treatments at the outpatient department (OPD) monthly. Officially the psychiatric clinic has 60 beds for inpatient services and 4 OPD.

### Participants

All patients with mental illnesses attending outpatient treatment at JMC were the source population of the study, while those who were available during data collection were the study population. Patients with mental illnesses who were age18 years and above were included whereas those who were acutely disturbed and unable to communicate well were excluded**.**

### Sample size determination

The sample size was determined by using the single population proportion formula, with the assumptions of 95% confidence interval, 5% marginal error, and the (p), the proportion of cluster B PDs to be 50%.

Where *n* = minimum required sample size

$$n=\frac{{\left(\frac{{\varvec{z}}\boldsymbol{\alpha }}{2}\right)}^{2}{\varvec{p}}{\varvec{q}}}{{{\varvec{d}}}^{2}} {\left(1.96\right)}^{2}{\mathrm{x}0.5\mathrm{ x }0.5/0.05}^{2}=0.9604/0.0025=384$$

By adding a 5% non-response rate, the final sample size was ***n* = 404.**

### Sampling techniques and procedures

Systematic random sampling was used to select the representative sample. The sampling interval was done by dividing the total number of patients visiting the outpatients within two months into the final sample size. K = 2000/404 = 5. Thus, the sample was selected every five intervals by using a registration book order. The registration book is a book in which every patient’s name and medical record numbers are recorded immediately after their arrival before they got any clinical service. The data was collected through face-to-face interviews by using pre-tested interviewer-administered questionnaires. Four BSc psychiatric professionals were employed for two months of data collection periods and supervised by one mental health specialist. Study participants were identified by data collectors by reviewing the patient’s registration book. Then, data was collected from selected study participants.

### Data collection instruments

The prevalence of cluster B personality disorder was measured using the personality diagnostic questionnaire (PDQ-4 +). It is a self-report tool with a true–false format; reflects a single DSM diagnostic criterion. Besides, a brief structured interview; the clinical significance scale, follows the self-report and either confirms or does not confirm the diagnosis for each PD scoring at/over the threshold. This interview directly reflects the principal DSM-IV/V general criteria for PDs assessing whether: (a) the trait is enduring (criterion D for DSM); (b) it is present in the absence of a psychopathological state, the effects of a substance, or any medical condition (criteria E and F); and (c) it leads to distress or impairment (criterion C) [24]. It has proven to have suitable psychometric properties both in its original version and in its adaptation to other languages and cultures, and clinical and non-clinical samples [25–29]. Its sensitivity ranges from 0.5 (histrionic PD) to 1 (antisocial PD) and specificity from 0.90 (borderline PD) to 0.98 (histrionic & narcissistic) [30] and diagnostic agreement (kappa) between PDQ-4 + and SCID-II was moderate (0.43) [25]. The reliability coefficient in this study was 0.93.

The level of social support was measured by the social support scale (Oslo-3). The social support scale (Oslo-3) was used to collect data regarding the strength of social support. The sum score is categorized into three broad categories of social support;3–8 poor social support, 9–11 moderate social support, and 12–14 strong social support [31]. The Cronbach’s alpha in this study was 0.83. The questionnaire has also covered a range of topics including socio-demographic factors, clinical factors, substance use, and risky behaviors (questions that assess suicidal thought (passive and active), suicidal attempt, homicidal thought, and attempt). Other mental illnesses diagnosis was obtained from the charts of the patients.

### Data quality control

The questionnaire was prepared first in English and translated into Afaan Oromo and Amharic by two of the authors and language experts with back translation to English by other language experts and mental health specialists who were not familiar with the purpose of the study. Some variations in language meaning and translation differences were resolved through a focus-group discussion. The training was given to four data collectors. A pre-test was conducted (5% of the sample size, *n* = 21) at Shenen Gibe general hospital which is around 15 km from JMC. Regular supervision and support were made for data collectors by the supervisor and principal investigator. Data were checked for completeness and consistency on daily basis during data collection time.

### Operational definitions

#### Personality disorder

If an individual fulfilled the diagnostic DSM-V threshold for specific PD through PDQ-4 measurement and confirmed by its clinical significance scale, the individual has a personality disorder [24].

#### Cluster B PD

If an individual was positive for at least one of four (borderline, antisocial, histrionic, and narcissistic) PDs [24].

#### Substance use

Ever (lifetime) and current( within past three months) use of any psychoactive substance [32].

#### Social support

The score of (3-8) poor, (9-11) moderate, and (12-14) strong social support on OSLO 3 scale (31).

### Data processing and analysis

Data were entered into Epi Data version 4.6 and analyzed by statistical package for social sciences (SPSS) version 26. Descriptive analysis was done using frequency, percentage, mean and standard deviation. The prevalence of self-reported PDQ-4 + scales was analyzed using the DSM-V thresholds. The clinical significance scale interview was confirming the diagnosis for screened-positive disorders, leading to dichotomous present/absent outcomes.

All variables were entered into a bivariate logistic regression to identify associated factors of cluster B PDs among people with a psychiatric disorder, and variables with a *p-value* < 0.25 were considered candidates for multivariable logistic regression analysis. In multivariable logistic regression analysis using a backward method, variables with a *p*-value less than 0.05 at a 95% confidence interval were considered statistically significant. Finally, the test for model fitness was done using the Hosmer–Lemeshow model test. The multicollinearity of the independent variables was checked by the variance inflation factor (VIF).

## Result

### Sociodemographic characteristics of respondents

Among 404 patients approached for an interview a total of 401 have participated in this study with a response rate of 99.3%. The mean age of the respondents was 34.69 (SD =  ± 10.94) years. Details of the demographic characteristics of study participants are presented in (Table 1).

**Table 1:** Socio-demographic characteristics of psychiatric outpatients in Jimma medical center, Southwest Ethiopia

Variable	Categories	Frequency	Percent
Sex	Male	256	63.8
	Female	145	36.2
Age	≥ 34.69	205	51.1
	< 34.69	196	48.9
Marital status	Single	197	49.1
	Married	143	35.7
	Divorced/ widowed	61	15.2
Educational status	College and above	189	46.6
	9-12th grade	143	35.7
	Unable to read & write	59	14.7
	1-8th grade	12	3.0
Occupational status	No	221	55.1
	Yes	180	44.9
Childhood care	Mother only	299	74.6
	father and mother	58	4.5
	Others^a^	44	10.9

^**a**^ Those who are out of father and mother

### Clinical related characteristics of respondents

Most of the study participants (40.1%, *N* = 161) had a diagnosis of major depressive disorder followed by schizophrenia (32.4%, *N* = 130). The mean duration of illnesses was 101.29(SD =  ± 73.4) months and the mean age onset of illnesses was 26.53 (SD =  ± 8.28) years. The mean duration of treatment was 86.92 (SD =  ± 75.4) months and the mean number of admissions was 1.39 (SD =  ± 0.49) times. The mean duration stayed in the hospital for those admitted was 1.25 (SD =  ± 0.44) months and the mean number of relapses was 1.54 (SD =  ± 0.49) times. More than one-third (35.4%, *N* = 142) of the respondents have a history of family mental illnesses.

### Substance use characteristic of respondents

The lifetime prevalence of alcohol, Khat, tobacco, and cannabis use among respondents was (16.2%, *N* = 65), (52.1%, *N* = 209), (16.5%, *N* = 66), and (6.2%, *N* = 25) respectively. About (8%, *N* = 32), (32%, *N* = 131), (8.5%, *N* = 34), and (9%, *N* = 36) of respondents were current users of alcohol, Khat, tobacco, and cannabis respectively, and more than half (57.9%, *N* = 71) of them started to use substance before age 17.

### Risky behaviors and social support status of respondents

Among study participants (47.1%, *N* = 189) had a history of passive suicidal thought, (33.9%, *N* = 136) active suicidal thought, (16%, *N* = 64) suicidal attempt, (19.2%, *N* = 77), homicidal thought, and (12.5%, *N* = 50) had a history of homicidal attempt in their life. Regarding the social support status of respondents (44.6%, *N* = 179) reported as they have poor social support according to the Oslo-3 social support scale measurement.

### Prevalence of cluster B personality disorder

Of all respondents, about 93(23.19%, 95%CI = 19 – 27) of them have cluster B personality disorder as measured by the PDQ-4 + with its clinical significance scale. The frequency of each cluster B personality disorders was 35(8.7%, 95%CI = 6–12), 29(7.2%, 95%CI = 5–10), 26(6.5%, 95%CI = 4–9), and 13(3.2%, 95%CI = 2–5) for borderline, antisocial, narcissistic and histrionic personality disorder respectively.

### Factors associated with cluster B personality disorders

From those candidate variables in bivariate analysis educational status, having a diagnosis of major depressive and bipolar-I disorder, duration of illness, number of relapses, family history of mental illness, suicidal attempt, current use of alcohol and cannabis, and having earlier age at substance-using started were found to be statistically significant.

Participants who can’t able to read and write were three (adjusted odds ratio (AOR) = 3.28, 95%CI = 1.43—7.51) times more likely to have cluster B personality disorder than those who have the educational status of college and above. Respondents who have no occupation is more than two (AOR = 2.32, 95%CI = 1.19—4.49) times more likely to have the disorder than their counterpart. Major depressive and bipolar-I disorder patients were near four (AOR = 3.72, 95%CI = 1.52–9.10) and almost three (AOR = 2.94, 95%CI = 1.37—6.29) times more likely to have cluster B personality disorders than schizophrenia patients respectively. Likewise, those patients who have a longer duration of illness (above the mean) and many relapses (above the mean) were more than two times more likely to have cluster B PDs than their counterparts (AOR = 2.44, 95%CI = 1.33—4.47) and (AOR = 2.21, 95%CI = 1.18—4.15) respectively. Those patients who have a family history of mental illnesses were two times more likely to have cluster B PD than those who have not (AOR = 2.05, 95%CI = 1.17—3.62), and also cluster B PD was more than two times more likely to present among those who have a history of suicidal attempt (AOR = 2.24, 95%CI = 1.01—4.96). Patients who use alcohol currently are almost three (AOR = 2.86, 95%CI = 1.34—6.10) times more likely than those who are not the user. Cluster B personality disorder is four times more likely to present among respondents who are using cannabis currently (AOR = 4.38, 95%CI = 1.61—11.95) and more than four (AOR = 4.42, 95%CI = 1.51 -12.96) times more likely to present among those who started to use substance earlier (before age 17) (Table 2).

**Table 2:** Bivariate and multivariable analysis of factors associated with cluster B personality disorders among psychiatric outpatients in Jimma medical center, Southwest Ethiopia

Variables	Cluster B personality disorders		OR (95%CI)		
	**Yes n (%)**	**No n (%)**	**Crude**	**Adjusted**	
Educational status					
College & above	36(19.3)	151(80.7)	1		1
9-12^th^ grade	35(24.5)	108(75.5)	1.35(0.80–2.30)		1.34 (0.72–2.51)
1-8^th^ grade	3(25)	9(75)	1.39(0.36–5.42)		2.43 (0.56–10.6)
Unable to read & write	19(32.2)	40(67.8)	1.99(1.03–3.84)		3.28 (1.43–7.52) ^a^
Occupational status					
Yes	26(14.5)	153(85.5)	1		1
No	67(30.2)	155(69.8)	2.54(1.54–4.21		2.32(1.19–4.49) ^a^
Psychiatric diagnosis					
Schizophrenia	21(16.2)	109(83.8)	1		1
Others psychotic d/o	2(25.3)	9(74.7)	1.15(0.23–5.72)		2.97 (0.53–16.77)
Bipolar-I disorders	25(25.3)	74(74.7)	1.75(0.96–3.36)		2.94 (1.37–6.30) ^a^
Major depression	45(28)	116(72)	2.01(1.13–3.59)		3.72 (1.52–9.10) ^a^
Mean duration of Illness (SD = ± 73.4)					
< 101.29 months	54(29.2)	131(70.8)	1		1
≥ 101.29 months	39(18.1)	177(81.9)	1.87(1.17–2.99)		2.44(1.33–4.47) ^a^
Mean number of relapses (SD = ± 0.49)					
< 1.54 times	27(14.7)	157(85.3)	1		1
≥ 1.54 times	66(30.4)	151(69.6)	2.91(1.74–4.83)		2.06(1.17–3.62) ^a^
History of family mental illness					
No	47(18.1)	212(81.9)	1		1
Yes	46(32.4)	96(67.6)	2.16(1.35–3.47)		2.24(1.01–4.96) ^a^
Suicidal attempt					
No	72(21.4)	265(78.6)	1		1
Yes	21(32.8)	43(67.2)	1.79(1.00–3.22)		2.24(1.01–4.96) ^a^
Alcohol(R)^b^					
No	81(22.0)	288(78.0)	1		1
Yes	12(37.5)	20(62.5)	2.13(1.00–4.54)		2.86(1.34–6.11) ^a^
Cannabis(R)^b^					
No	80(21.9)	285(78.1)	1		1
Yes	13(36.1)	23(63.9)	2.4(0.96–4.15)		4.38(1.61–11.95) ^a^
Mean age at substance using started (SD = ± 9.46)					
≥ 17.79 years	9(11.7)	68(88.3)	1		1
< 17.79 years	84(25.9)	240(74.1)	0.38(0.18–0.79)		4.42(1.51–12.96) ^a^

^a^ Significant variables on multivariable analysis

R^b^ Current use of the substance, 1: Reference group

## Discussion

In this study out of the total respondents, the prevalence of cluster B PD was found to be 93(23.19%, 95%CI = 19–27). The finding is in agreement with a study conducted in Turkey, Netherland, and Italy which reported the prevalence of cluster B PD was 23.2%, 24.5%, and 25.8% respectively [33–35].

The figure is higher than the studies conducted in Kenya, Rhode Island, and China which revealed that the prevalence of cluster B PD was 17.6%, 13%, and 9.8% respectively [9, 16, 36]. The difference might be due to the difference in the instrument used in which structured clinical interview is used in those studies, a tool which is known for its low-frequency report compared to self-report screening tools like ours (PDQ-4 +). The other issue that might explain the disparity is the study population, in which the study was conducted among admitted patients in Kenya, and a different set of studies, from psychiatric and psycho counseling clinics, was used in China in contrast to ours which was only from the psychiatric outpatient department.

The frequency of our study was found to be lower than the study conducted in Canada and Oxford which reported the prevalence of cluster B PDs was 32% and 28.8% respectively [37, 38]. The difference in prevalence is likely to be due to differences in participants of the study in which only alcohol use disorder patients in the Canada study and deliberate self-harm patients in the Oxford study participated. It might be also due to the difference in the tool used, which structured interviews for DSM-IV PDs in Canada, and the personality assessment schedule in Oxford was used.

Borderline PD was found to be two times more prevalent among females and antisocial was three times more prevalent among males, while there was no significant difference between histrionic and narcissistic PDs in terms of sex. This is in agreement with a study conducted in Kenya among admitted patients [9]. Among the respondents 4(1%) of them have borderline and antisocial, 2(0.5%) borderline and histrionic, and 2(0.5%) borderline and narcissistic personality disorders. This indicated borderline PD was found to be comorbid with all other disorders within the cluster, which is supported by different studies conducted in different countries which documented that borderline PD was the most comorbid disorder with other PDs [39, 40].

Regarding associated factors of cluster B PD, those respondents who can’t read and write were more than three times more likely to have the disorder than those who attended college and above. The finding is in line with a study from the USA which reported that educational status is inversely related to cluster B PDs [7]. Refusal of going to school, early dropout, and low educational attainment among those with cluster B PD could be the explanation. Those respondents who have no occupation are more than two times more likely to have the disorder than those who have an occupation. The finding is consistent with a study from the USA which reports the positive relationship between unemployment and the disorder [7]. In this study cluster B PD was almost four and nearly three times more likely to present among major depressive and bipolar-I disorder patients respectively than schizophrenia patients. The finding is supported by a study from Kenya which stated that mood disorder was the most comorbid with PD (46%) [9] and a study conducted in China which revealed that cluster B PD was more common among patients with affective disorders(12.2%) than schizophrenia patients(3.8%) [10]. Those respondents who have a longer duration of illness (101.3 months and above) were more than two times more likely to have the disorder than their counterparts. The earlier onset of symptoms, obstacles to treatment like non-adherence due to interpersonal functioning impairment, and poorer response to treatment among those who have comorbid PD could be the reason [35, 41–43].

In this study, the disorder was more than two times more likely to present among the respondents who have multiple relapses. The finding is in agreement with the studies from France and Dutch which revealed that those patients who have comorbid PDs experienced more relapses than those who do not [11, 44]. The respondents who have a history of family mental illness were two times more likely to have cluster B PD than those who have not. This is similar to the study from Kenya that explained a family history of mental illness was significantly associated with positive and negative scores of PD [9]. The reason might be almost all psychiatric illnesses including personality disorders are genetically influenced and run around the family [21].

The disorder was more than two times more likely to present among participants who have a history of suicidal attempts than their counterparts. The finding is supported by the study conducted in Japan which reported a greater number of suicidal attempts among cluster B PD [45] and a study from Oxford that explained suicidal attempts to be more common among depressed patients with comorbid borderline PD than depressive patients without comorbidity [37]. It is also supported by the multisite collaborative longitudinal study which reported that 12.5% of respondents who have the disorder attempted suicide within three years of follow-up [46] and a study conducted in Turkey which documented that a history of suicide attempts was significantly common in patients comorbid with any cluster B personality disorders [35]. Even though it was not associated with the prevalence of cluster B PDs the frequency of homicidal attempts was high (12.5%) in this study which was not a common report in the outpatient department. This might be due to the fact that JMC gives services including for prisoners from Jimma zonal correctional institute since there was no psychiatric clinic that gives services for the patients in the compound unlike in developed countries. However, at the beginning of 2022(after this study was conducted) the psychiatric clinic is established in the correctional institute after long-time efforts of Jimma University, psychiatry department staff.

Current users of alcohol were almost three times more likely to have the disorder than those who are not the user. It is in agreement with studies conducted in Japan and the USA which documented a high frequency of alcoholism among those with cluster B PDs [7, 45]. The disorder was more than four times more likely to be found among those participants who are current users of cannabis. The finding is supported by a study from Kenya which reported that there was a significant association between cluster B PDs and cannabis use (31%) [9], a study conducted in Connecticut southeastern USA, reported the highest rate of recent cannabis use among individuals with PDs [47] and study from Turkey which revealed the frequency of cannabis among the participants with the disorder is 67% [48]. Additionally, our finding indicated that those participants who started to use substances at an earlier age (before age 17) are more than four times more likely to have the disorder than those who started later. The reason might be due to common etiologic processes with early expression of impaired impulse control and affective dysregulation [49]. The age of onset of the personality disorders, which is in adolescence/early adulthood the time at independence from family/caregiver and trying new events like substance use despite its consequences are exercised, the type of defense mechanism used by this group that is most of the time acting out and their inability to conform to the social norms might be the other reasons [21]. The high amount ((52.1%) respondents of this study were Khat users, even if it is not significantly associated (*p*-value = 0.15, AOR = 1.96, 95%CI = 0.79- 4.84) with the prevalence of cluster B PD. This high frequency could be due to the fact that Southwest Ethiopia is one of the areas where Khat plant is broadly grown and the Khat chewing is highly encouraged culturally.

### Limitations

It is important to note that there are some limitations to this study. Firstly, the PDQ-4 + was not validated in the language of the study population, even though we found it sufficient after a pretest. Additionally, some questions like that asked for childhood care giver was assessed retrospectively several years backward which there is chance of recall bias. The other limitation of the study is the severity of substance use was not assessed.

## Conclusion

This study revealed that the prevalence of cluster B personality disorders was high among mentally ill outpatients. The presence of the diagnosis of mood disorders, longer duration of illness, multiple relapses, family history of mental illness, history of suicidal attempts, recent use of alcohol and cannabis, and early age at substance use started were significantly associated with cluster B PDs. Therefore, it is important to give more emphasis to screening comorbid cluster B PDs as daily routine activities to give PD-oriented psychotherapy and engage and retain these patients in treatment, which helps to improve the course and treatment of the other disorder that patients typically identify as their chief complaint.

## Data Availability

The datasets used for analysis in this study are not publicly available due to the privacy of our respondents, and it contains additional information which was not included in this paper but is available from the corresponding author on reasonable request.

## Abbreviations

AOR: Adjusted odds ratio
COR: Crude odds ratio
DSM: Diagnostic and statistical manual of psychiatry
JMC: Jimma medical center
OPD: Outpatient department
PDs: Personality disorders
PDQ-4 +: Personality disorders questionnaire four-plus
SCID-II: Structured clinical interview for DSM-IV personality disorder
